# Potential of nanoformulations in malaria treatment

**DOI:** 10.3389/fphar.2022.999300

**Published:** 2022-10-26

**Authors:** Janaina Braga Chaves, Bianca Portugal Tavares de Moraes, Stela Regina Ferrarini, Francisco Noé da Fonseca, Adriana Ribeiro Silva, Cassiano Felippe Gonçalves-de-Albuquerque

**Affiliations:** ^1^ Immunopharmacology Laboratory, Department of Biochemistry, Federal University of the State of Rio de Janeiro—UNIRIO, Rio de Janeiro, Brazil; ^2^ Pharmaceutical Nanotechnology Laboratory, Federal University of Mato Grosso of Sinop Campus—UFMT, Cuiabá, Brazil; ^3^ Empresa Brasileira de Pesquisa Agropecuária, Parque Estação Biológica—PqEB, EMBRAPA, Brasília, Brazil; ^4^ Immunopharmacology Laboratory, Oswaldo Cruz Foundation, FIOCRUZ—UNIRIO, Rio de Janeiro, Brazil

**Keywords:** malaria, treatment, pre-clinical study, nanotechnology, infectious disease

## Abstract

Malaria is caused by the protozoan *Plasmodium sp* and affects millions of people worldwide. Its clinical form ranges from asymptomatic to potentially fatal and severe. Current treatments include single drugs such as chloroquine, lumefantrine, primaquine, or in combination with artemisinin or its derivatives. Resistance to antimalarial drugs has increased; therefore, there is an urgent need to diversify therapeutic approaches. The disease cycle is influenced by biological, social, and anthropological factors. This longevity and complexity contributes to the records of drug resistance, where further studies and proposals for new therapeutic formulations are needed for successful treatment of malaria. Nanotechnology is promising for drug development. Preclinical formulations with antimalarial agents have shown positive results, but only a few have progressed to clinical phase. Therefore, studies focusing on the development and evaluation of antimalarial formulations should be encouraged because of their enormous therapeutic potential.

## Introduction

Malaria is a disease that affected 241 million people and led to 627,000 deaths worldwide in 2020. It is considered a significant public health problem that preferentially occurs in tropical and subtropical regions and is an endemic disease in 85 countries (World malaria report, 2021).

The incidence of malaria occurs in an environment conducive to the spread of the vector mosquito, geographically in developing and underdeveloped countries. Among the 85 countries reporting malaria cases in 2020, 29 accounted for 96% of malaria cases worldwide, and six countries in the African continent accounted for 55% of global malaria incidence. Malaria mortality rate (deaths per 100,000 inhabitants at risk) decreased from 30% in 2000 to 15% in 2015 and to 13% in 2019. However, in 2020, the mortality rate increased to 15%. The increased mortality rate of malaria in 2020 was associated with the interruption of medical services for malaria treatment due to COVID-19 pandemic (World malaria report, 2021).

**FIGURE 1:**
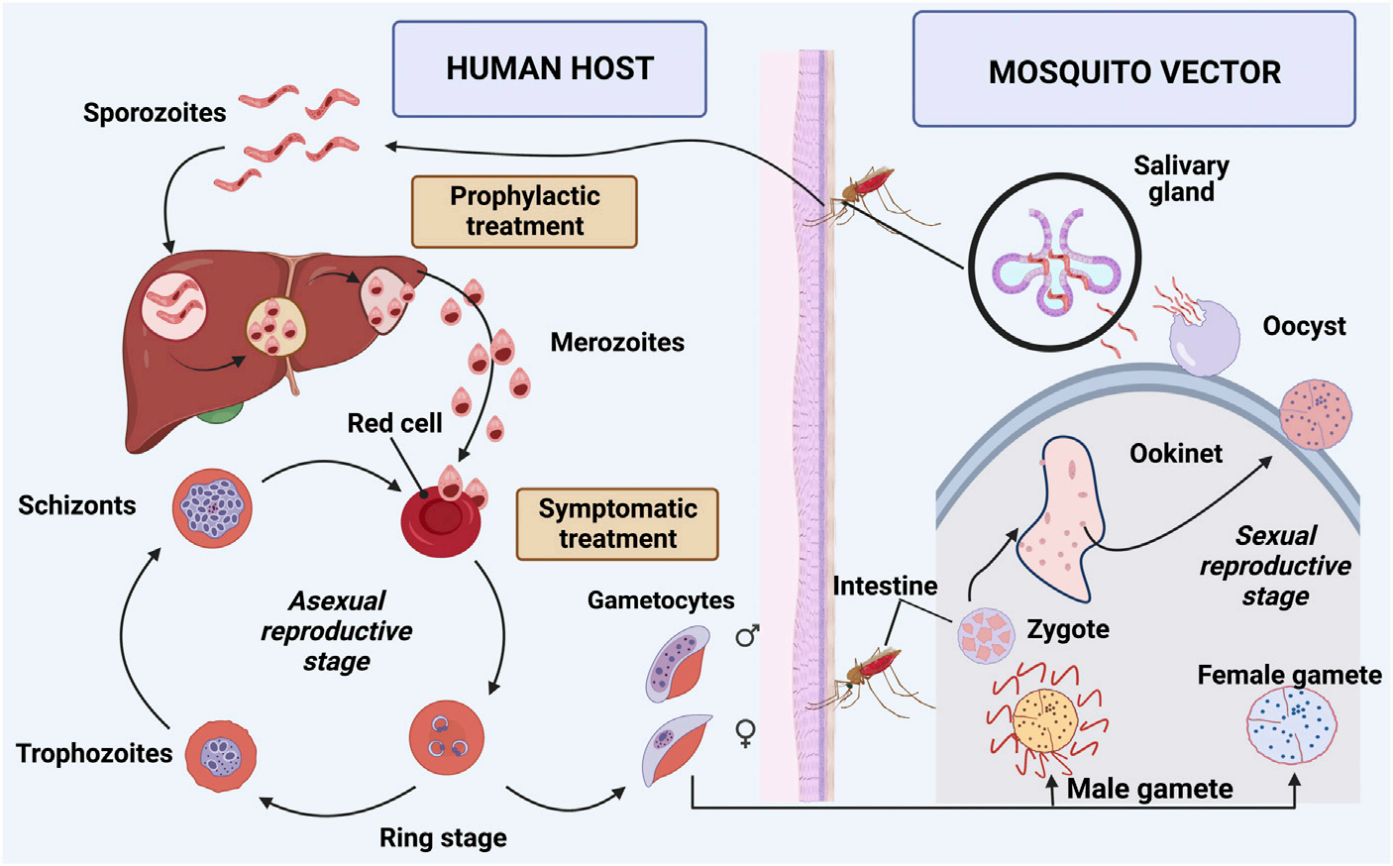
Life cycle of *Plasmodium* sp. The cycle can be divided into two stages: mosquito vector or sexual cycle and human host or asexual cycle. The mosquito ingests gametocytes while performing hematophagy. The zygote is formed from the union of gametocytes and generates oocyte. It crosses the intestinal wall and forms oocyst that releases sporozoites, which migrate to the mosquito’s salivary glands, completing the sexual cycle. The infected female *Anopheles sp* mosquito inoculates sporozoites, performs hematophagy, and begins the asexual cycle of *Plasmodium sp* in human. Sporozoites are transported to the liver through the blood, and asexual multiplication occurs in the hepatocytes, forming merozoites in the pre-erythrocytic cycle (Ashley et al., 2018). In *P. vivax* and *P. ovale* infections, some sporozoites differentiate in the liver to a latent form called hypnozoites. After rupture of the hepatocytes, merozoites are released into the bloodstream and penetrate the erythrocytes (erythrocyte phase), assuming a ring-shaped configuration (Coban et al., 2018). Proliferative schizogony occurs in infected erythrocytes, where merozoites multiply asexually, differentiating into schizonts and trophozoites. Erythrocytes rupture and release schizonts into the bloodstream, where one part differentiates into male and female gametocytes, and another part infects new erythrocytes (Phillips et al., 2017; Ashley et al., 2018). Image was created in BioRender.com.

The causative agent of malaria is the protozoan *Plasmodium sp*, which is inoculated into the human body by mosquitoes of the genus *Anopheles* during hematophagy. There is a complex mosquito-human-parasite cycle, and five species of the parasite can infect humans: *Plasmodium falciparum, Plasmodium vivax, Plasmodium ovale, Plasmodium malariae, and Plasmodium knowlesi* (Phillips et al., 2017; Ashley et al., 2018). Clinical manifestation of the disease in humans occurs due to the pre-programmed biology of the parasite in conjunction with the human pathophysiological response (Milner, 2018; Knackstedt et al., 2019). Two distinct stages occur in the life cycle of *Plasmodium sp*: sexual cycle in the vector mosquito and asexual cycle in the human host (Figure 1).

## Pathophysiology

The pathogenesis of malaria is related to the blood cycle. Symptoms usually appear approximately 10–15 days after infection, and the disease evolves with febrile response and potentially progresses to severe malaria, which is a consequence of parasite multiplication and invasion of red blood cells by parasites (Najer et al., 2018; Knackstedt et al., 2019). Malaria can be classified as asymptomatic, uncomplicated, and severe (World malaria report, 2020). Any *Plasmodium sp* can cause asymptomatic malaria (Brazier et al., 2017) or uncomplicated malaria, which is manifested as chills, sweating, headache, nausea, or vomiting without severe organ dysfunction (Phillips et al., 2017; Moxon et al., 2020). *P. falciparum* causes the most severe malaria disease, with severe organ damage, anemia, and hyperparasitemia (Brazier et al., 2017; Moxon et al., 2020).

Disruption of *Plasmodium sp*-infected erythrocytes leads to the release of malaria parasites and endotoxins, a complex of parasite DNA and hemozoin (Brazier et al., 2017; Ashley et al., 2018). Endotoxins are recognized by immune cells through Toll-like receptor 9 (TLR9), which increases the production of cytokines and chemokines (Karunaweera et al., 1992; Mubaraki et al., 2017). Oxidative stress increases the inflammatory response by releasing cytokines that cause organ damage (Dunst et al., 2017; Katsoulis et al., 2021). The membrane of erythrocytes infected by parasites hardens and loses its standard shape, contributing to the obstruction of capillaries and thrombus formation. Consequently, when vital organs are affected, severe malaria progresses to death (Coban et al., 2018; Klinkhamhom et al., 2020).

### Malaria treatment recommended by World Health Organization

The first- and second-line of treatment recommended by the World Health Organization (WHO) for uncomplicated *P. falciparum* malaria and chloroquine-resistant *P. vivax* is artemisinin-based combination therapies (ACTs) (Report on antimalarial drug efficacy, 2020; World malaria report, 2020). This therapy combines an artemisinin derivative with a partner drug. Artemisinin compound plays an important role in reducing the number of parasites during the first 3 days of treatment. After this period, the partner drug eliminates the remaining parasites (Report on antimalarial drug efficacy, 2020).

**FIGURE 2:**
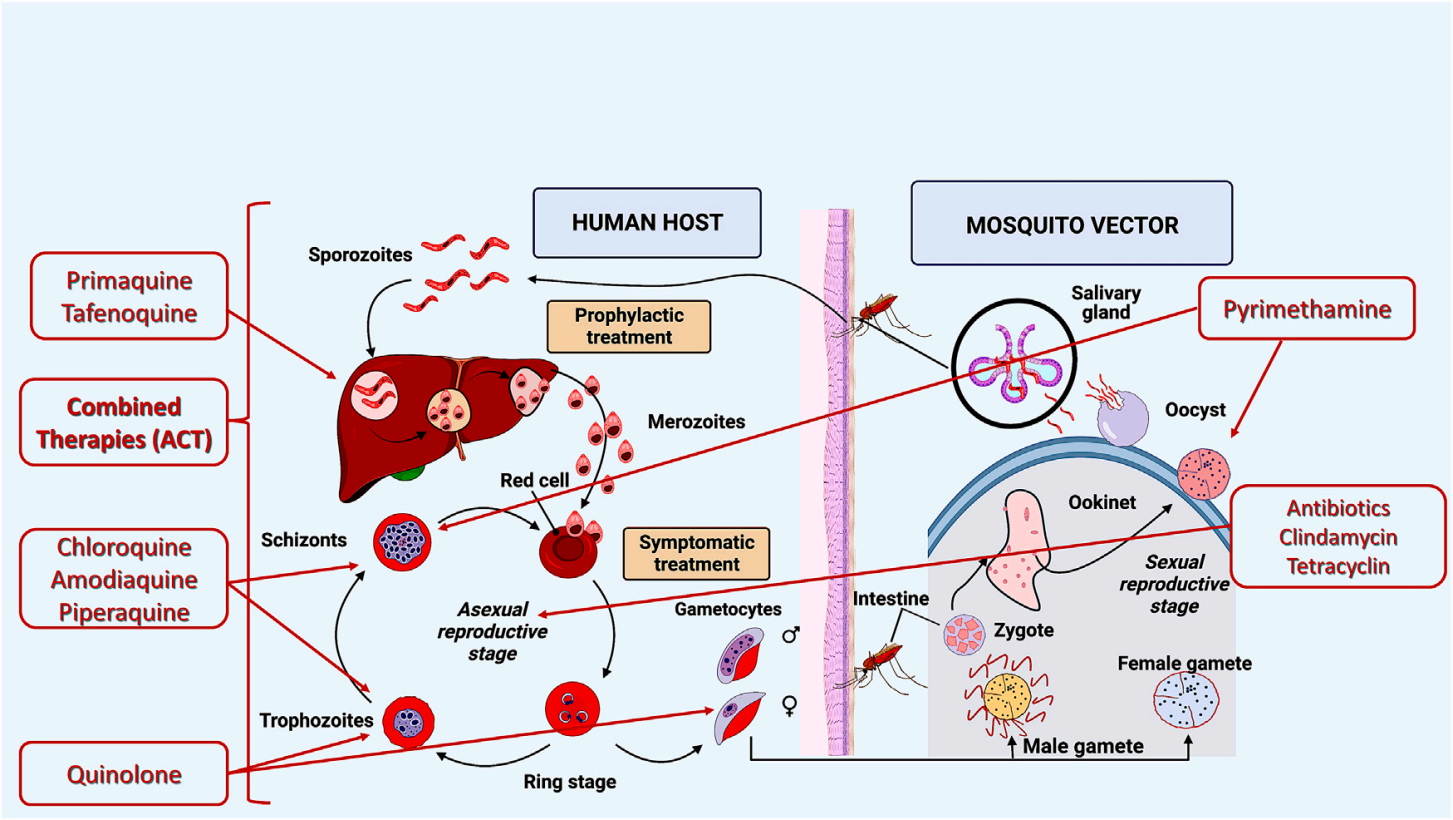
Current drug targets for malaria treatment: I—Asexual cycle; II—Liver cycle, III, IV, VI—Erythrocytic cycle, V—Asexual and sexual cycles. Source: Artemisinin-based combination therapies (ACTs) are recommended by the World Health Organization (World malaria report, 2020; Wicht et al., 2020; World malaria report, 2021).

Currently, several drugs that act during different stages of the parasite’s biological cycle are available for malaria prevention and cure (Figure 2). Most antimalarial agents target erythrocytic and asexual stages (Belete, 2020; Madhav and Hoda, 2021). Tafenoquine and primaquine are approved antimalarial agents against parasites and hypnozoites at the hepatic stage (Thriemer et al., 2017; Commons Id et al., 2019).

The WHO currently recommends six ACTs as the first- and second-line of treatment for uncomplicated cases (Table 1). In regions where malaria transmission is moderate to high, the WHO recommends intermittent preventive treatment in pregnancy (IPTp) during consultation from the beginning of the second trimester of pregnancy (Table 2) (Report on antimalarial drug efficacy, 2020).

**TABLE 1:** WHO-recommended artemisinin-based combination therapies (ACTs).

*P. falciparum* (uncomplicated)	*P. vivax* (uncomplicated)
Artemether-lumefantrine (AL)	Chloroquine (CQ)
Artesunate-amodiaquine (AS-AQ)	ACTs in areas with CQ resistance
Artesunate-mefloquine (AS-MQ)	
Artesunate-pyronaridine (AS-PY)	
Artesunate + sulfadoxine-pyrimethamine (AS + SP)	
Dihydroartemisinin-piperaquine (DHA-PPQ)	

Source: Adapted from Report on Antimalarial Drug Efficacy, Resistance and Response: 10 years of Surveillance (Report on antimalarial drug efficacy, 2020; World malaria report, 2020).

**TABLE 2:** WHO-recommended treatment for uncomplicated malaria in pregnant woman.

*P. falciparum*		
Not complicated		ACT
First, second, and third-trimester of pregnancy		Quinine + clindamycin ACT
*P. vivax, P. ovale, P. malarie, P. knowlesi*		
Blood stage	Not complicated	Chloroquine or ACT
	1st-trimester pregnant women	Chloroquine or quinine
	2nd trimester	Chloroquine or ACT
Liver stage ( *P. ovale/P. vivax* )		Primaquine^a^

Source: Adapted from Report on Antimalarial Drug Efficacy, Resistance and Response: 10 years of Surveillance (Report on antimalarial drug efficacy, 2020; World malaria report, 2020).

^a^ Not recommended for children <6 months, pregnant women, nursing mothers, or those with glucose-6-phosphate dehydrogenase (G6PD) deficiency.

## Resistance to antimalarials

Over the years, several endemic areas worldwide have reported increased incidence of *Plasmodium* drug resistance. Increasing drug resistance of *Plasmodium sp* is one of the main factors responsible for treatment failure (Rai et al., 2017; L-QuraishAy et al., 2020). In addition, the use of drugs from the same chemical family or having a similar mode of action may have intensified cross-resistance to antimalarial drugs (Capela et al., 2019; Tse et al., 2019).

Several studies have reported that molecular mechanisms of resistance to antimalarial drugs occur in several species of parasites and include polymorphisms in proteins that alter the physiological regulation in the parasite (Menard and Dondorp, 2017; Wicht et al., 2020). The occurrence of polymorphisms makes it obvious that resistance to antimalarial drugs is associated with the genetic factors of parasites. Single, double, or quadruple mutations in different genes confer resistance in the parasite to antimalarial drugs. For example, mutations in the *Pfmdr1, Pfcrt, Pfmrp,* and *Pfnhe1* genes confer drug resistance (Cubides et al., 2018; WWARN K13 Genotype-Phenotype Study Group, 2019).

Mutations in the *Pfmdr1* membrane transporter found in *Plasmodium sp* digestive vacuoles may influence parasite susceptibility to chloroquine (Phillips et al., 2017; Bree and levy, 2018). The *Pfcrt* and *Pfmdr1* are vital multidrug resistance proteins. The *Pfmdr1* encodes a p-glycoprotein homolog that affects sensitivity to multiple antimalarial drugs (Ross et al., 2018; Xu et al., 2018) including artemisinin, mefloquine, lumefantrine, quinine, and chloroquine (Patel et al., 2017; Xu et al., 2018). Atovaquone is a synthetic hydroxynaphthoquinone with antiprotozoal activity. Atovaquone interferes with DNA synthesis by blocking mitochondrial transport of electrons from the protozoan respiratory chain, leading to cell death (Staines et al., 2018; Kathpalia et al., 2020). Unique mutations in the P*fcytb* gene of *P. falciparum* caused resistance to atovaquone in *in vitro/in vivo* experiments (Staines et al., 2018; Kathpalia et al., 2020). The gene encoding *P. falciparum* Kelch 13 (*PfKelch13*) has been identified as a genetic determinant of resistance (Bree and levy, 2018; WWARN K13 Genotype-Phenotype Study Group, 2019). K13 mutations reduce protein function and cause artemisinin resistance by reducing its activation (Talman et al., 2019; Wicht et al., 2020).

All molecular targets of antimalarial drugs have not been defined. Drug resistance can occur via several pathways, such as processes that reduce drug toxicity, some catalytic processes that promote changes in enzyme reactions, or amplification of the gene encoding the target enzyme or transporter that pumps the drug out of the parasite (Ross et al., 2018; Deda et al., 2020). Surveillance of resistance to antimalarials is performed using three complementary approaches: 1) *in vivo* studies to evaluate the efficacy of medications in patients, 2) *in vitro* studies to assess the parasite susceptibility to medicinal products, and 3) molecular studies to detect genetic mutations and/or gene copy number alterations associated with drug resistance (Xu et al., 2018; Nsanzabana, 2019; Report on antimalarial drug efficacy, 2020). Many studies have described factors that indicate resistance to most drugs used to treat malaria and reveal possible targets for new drugs. With the advancement of molecular biology, metabolomics and proteomics details of the parasite support the development of new pharmacological agents such as nanopharmaceuticals. The work between academic research and pharmaceutical industry is essential and positive for treating malaria cases in endemic regions with efficient and technically targeted approaches.

### Disadvantages of conventional antimalarials

The most evident disadvantage of conventional antimalarial drugs is *Plasmodium sp* resistance due to genetic polymorphisms (Bree and levy, 2018; Wicht et al., 2020). However, other disadvantages that influence malaria treatment with conventional antimalarial drugs include low water solubility, low bioavailability, side effects, and relatively short half-life (Alven and Aderibigbe, 2020; Rashidzadeh et al., 2021; Souza Botelho et al., 2021). Side effects frequently related to conventional antimalarial drugs include abdominal pain and gastrointestinal symptoms such as vomiting, jaundice, itching, hypoglycemia, anemia, dizziness, coma, and altered consciousness (Novitt-Moreno et al., 2021; Rashidzadeh et al., 2021). In addition, during prolonged use, there is a risk of hemolysis (tafenoquine and primaquine), retinopathy, mental confusion, cardiac complications (tafenoquine and chloroquine) (Novitt-Moreno et al., 2021), and gastric irritation (primaquine) (da Silva de Barros et al., 2021). Skin hypersensitivity reactions to sulfadoxine-pyrimethamine (Stevens-Johnson syndrome), severe hepatoxicity, and neuropsychiatric reactions to mefloquine have also been reported (Frey et al., 2010; Ashley and Phyo, 2018). In many cases, serious side effects resulted in treatment discontinuation (Brito-Sousa et al., 2019; Who, 2019).

### Development of new drugs/pharmaceuticals

Metabolic pathways, such as nucleic acid synthesis, heme detoxification, oxidative fatty acid synthesis, and stress, are the primary targets for development of new drugs (Baruah et al., 2017; Oyelade et al., 2019). In the search for new treatments, pharmaceutical companies have studied a variety of drug candidates for malaria control and elimination (Belete, 2020). New agents such as arterolane, cipargamin, and KAF156 have the potential to replace ACTs that fail to treat malaria infection. Therefore, there is an urgent need to reassess the current combination therapy for malaria treatment (Ashley and Phyo, 2018; Moyo et al., 2020).

To develop antiparasitic molecules, phenotypic screening studies of the parasite are essential (Cowell and Winzeler, 2019; Yahiya et al., 2019). In addition, phenotypic screening for the entire biological cycle of *Plasmodium* sp is needed to gather data in chemical libraries and enable discovery of multiple substances that have potential as antimalarials (Cowell and Winzeler, 2019; Yahiya et al., 2019).

Kae609, KAF156, DSM265, and MMV048 are the four most advanced antimalarials have emerged from multidisciplinary collaboration and are currently in phase II trials. The main objective of malaria treatment and elimination strategies is to target multiple stages of the parasite cycle (Summers et al., 2021). Combined antimalarial treatments that do not present artemisinin (ART) are recommended by the WHO when unavailable or suitable for treatment (Who, 2015). An open randomized phase III clinical study compared the efficacy of quinine/clindamycin with artemether/lumefantrine in treating uncomplicated malaria in children below 5 years of age and did not find evidence for the use of quinine/clindamycin when artemether/lumefantrine is still effective (Obonyo et al., 2022). A single monthly prophylactic antimalarial drug composed of a combination of naphthoquinone-azithromycin (NQAZ) was used in a randomized, placebo-controlled, double-blind study to evaluate its protective effect against *Plasmodium* infection. Treatment with NQAZ showed 93.62% protective efficacy with a 95% confidence interval [CI] of 91.72–95.52 (Yang et al., 2021). A two-group, multicenter, and randomized comparative study compared the efficacy of a dispersible tablet composed of a combination of a fixed dose of arterolane maleate (AM) 37.5 mg and piperaquine phosphate (PQP) 187.5 mg and that of artemether-lumefantrine (AL) in pediatric patients with *P. falciparum* infection. Both treatments were considered safe with good tolerance, and the efficacy of the AM-PQP combination was compared to that of AL (Toure et al., 2016). There is an urgent need to develop rapid action antimalarials that act during the asexual stage in the blood to reduce the propensity to generate resistance. The four most successful antimalarials primarily target multiple stages of the malaria parasite’s life cycle (Ashton et al., 2019).

### Potential of nanotechnology in malaria

**FIGURE 3:**
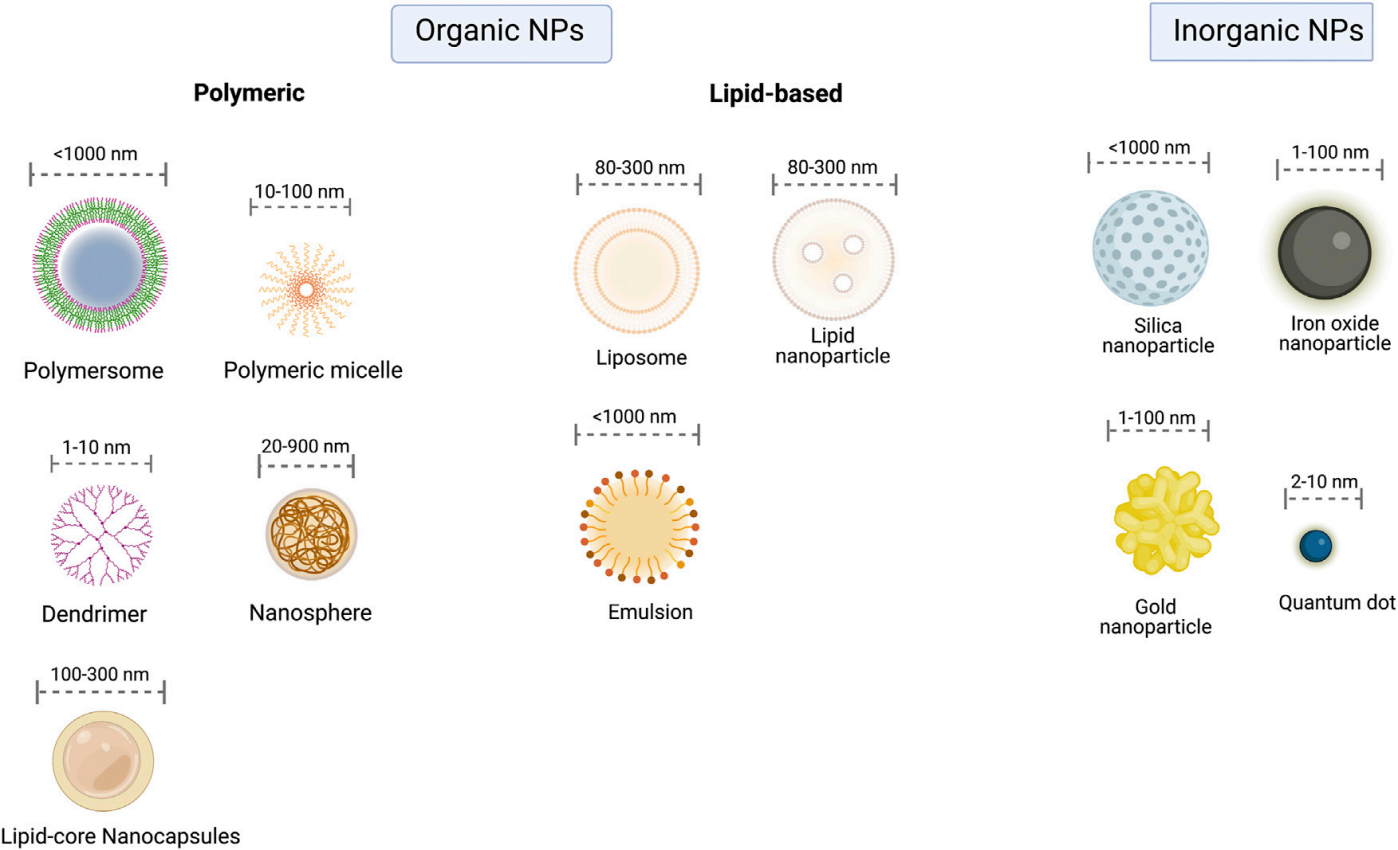
Classes of Nanoparticles. Nanoparticles can be divided into organic (lipid-based and polymeric) and inorganic. Each class embodies several NPs with the most relevant highlighted in the figure.

Recent studies have demonstrated the potential of nanotechnology for the treatment of different diseases through multiple techniques (Figure 3). Nanostructured drug delivery systems have clinical applications in the treatment of immunological disorders such as allergy, cancer, arteriosclerosis, diabetes, and malaria (Calderó et al., 2017; Charlie-Silva et al., 2018). FDA have already approved nanotherapies for a variety of applications, but at the best of our knowledge, none yet for malaria treatment (Mitchell et al., 2021).

A considerable improvement in the pharmacokinetics and efficiency of the encapsulated nano drug was observed compared with that of the encapsulated free drug (Abdolmaleki et al., 2021; Biosca et al., 2021). Critical studies based on nanotechnology for the development of antimalarial drugs are aimed to solve key problem in malaria treatment, such as disease severity, and are focused on reduced level of drug toxicity, interruption of transmission of *Plasmodium sp*, increased efficacy of drugs, and mainly, combating multidrug resistance (Manconi et al., 2019; Nnamani et al., 2021). The use of nanostructured drug delivery systems can potentially solve the critical issue of *Plasmodium sp* multidrug resistance to drugs used for a long time in endemic regions, offering a new possibility of using the same drugs at manometric concentrations with reduced side effects (Marwah et al., 2020; Elmi et al., 2021).

**FIGURE 4:**
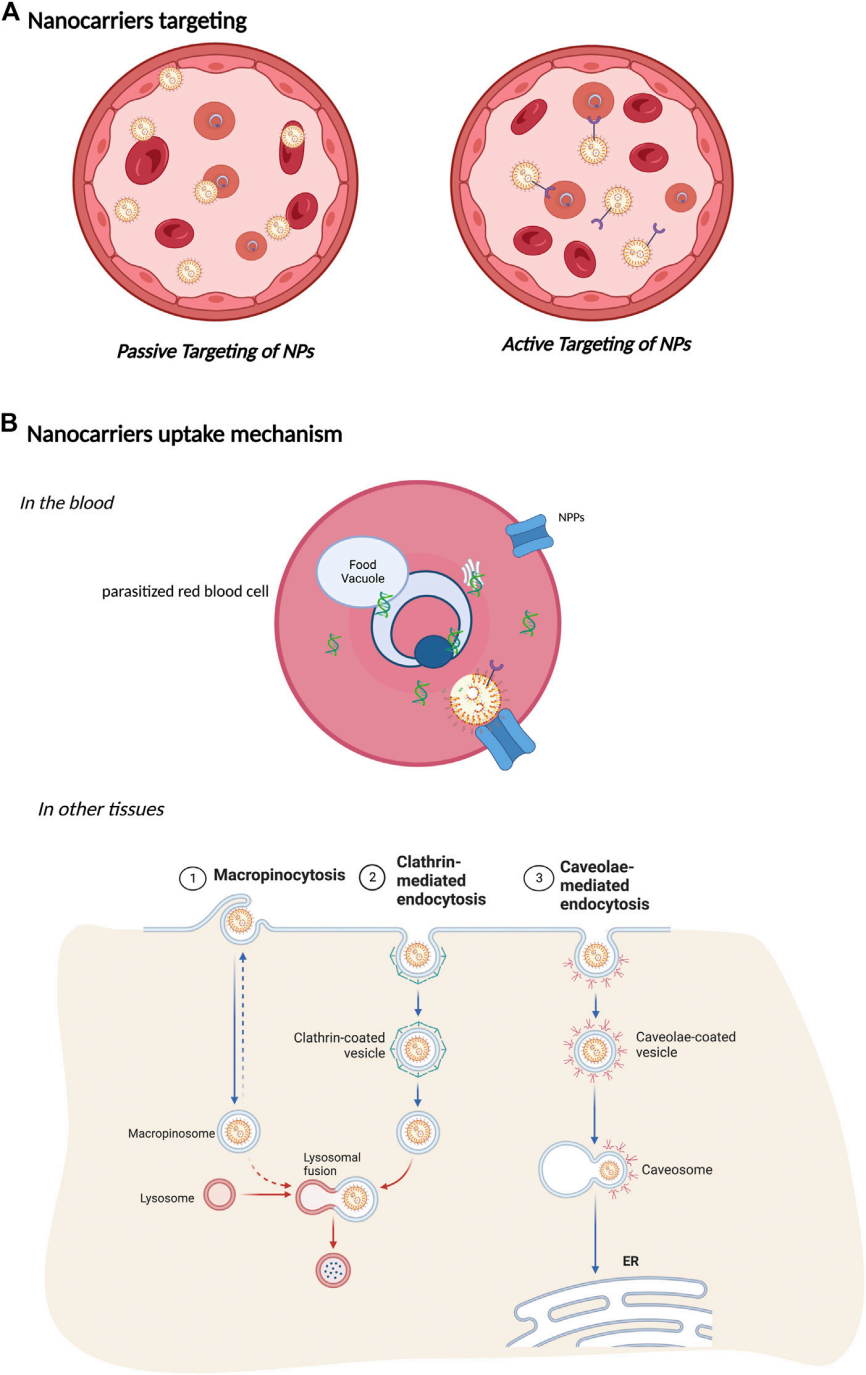
Nanocarriers. **(A)** Nanocarriers targeting. A schematic diagram represents the active and passive targeting of NPs. In passive targeting, NPs are carried by red blood cells and in the bloodstream to all tissues. During active targeting, NPs are conjugated with affinity ligands on their surface to enhance their uptake by the target site and cells. Different targeting moieties, such as antibodies, integrins, folate, glucose, or transferrin, can interact with molecules on the target cell surface. **(B)** Nanocarrier uptake mechanism. In the blood, nanocarriers can be targeted to recognize only parasitized red blood cells. These reduce the chances of resistant pathogen strains and side effects for the patient since the intake is considerably low compared to traditional treatments. In addition, Plasmodium induces new permeation pathways (NPPs) that confer increased permeability to the pRBC by changing the osmotic stability and enabling nanocarriers to enter the pRBC. In other tissues, intracellular uptake of nanocarriers follows different endocytosis pathways. When nanocarriers reach the cell surface, they are taken up by endocytosis depending on their shape, charge, size, or surface. Endocytosis can occur by macropinocytosis, driven by membrane ruffling and actin protrusions. After engulfment, they fuse with lysosomal compartments to content degradation. Clathrin-mediated endocytosis is based on clathrin-coated pits on the cytosolic side of the membrane forming clathrin-coated vesicles that undergo vesicular trafficking. Caveolae-mediated endocytosis undergoes the same dynamics. However, they fuse to caveosomes, avoiding lysosomal degradation.

Targeting a drug to unveil its precise mechanism of action is a crucial strategy for treating malaria. Several barriers must be eliminated to allow the drug to reach the intracellular parasite. Bioavailability, concentration, and elimination of drugs are important factors that need to be considered for successful treatment. Nanopharmaceuticals have a promising prospect (Panzarini et al., 2018; Akpa et al., 2020; Pestehchian et al., 2020; Zhang et al., 2020; Guo et al., 2021; Wang et al., 2021). The application of nano-based delivery systems as carriers of antimalarial drugs has resolved some essential issues, such as increased therapeutic effect of conventional antimalarials with decreased resistance of *Plasmodium* sp and selective distribution of drugs (Abazari et al., 2020; Rashidzadeh et al., 2021) (Figure 4).

Cerebral malaria is considered to be extremely severe, which is manifested by intense inflammatory conditions and severe organ damage. The drugs used for the treatment of cerebral malaria are generally administered intravenously; therefore, drug concentration and side effects are the major issues that hinder the treatment of patients with cerebral malaria. Nanostructured delivery systems can potentially treat malaria with less harm to patient (Golenser et al., 2020; Agbo et al., 2021). Different polymer-based nanoparticle structures, such as drug delivery systems, have been studied and improved in multiple studies (Nosrati et al., 2018; Ramazani et al., 2018; Abazari et al., 2020; Dias et al., 2020).

*In vitro* and *in vivo* studies used the Pfs25 sex stage gene from *P. falciparum*, harmonized by codon in *Escherichia coli* (CHrPfs25), as antigen conjugated to the gold nanoparticle (GN) in different sizes and shapes. GNs can act as a vaccine delivery vehicle because they strongly induce antibodies that block *P. falciparum* transmission. Authors found IgG from mice immunized with different GN-particles with highly potent blocking activity regardless of IgG isotype differences (Kumar et al., 2015).

A DNA vaccine study was carried out using magnetic vectors composed of superparamagnetic iron oxide nanoparticles (SPIONs), hyaluronic acid (HA), and polyethyleneimine (PEI) added to the DNA encoding PyMSP1 19 of *Plasmodium yoelii*. The complex induced cellular and humoral immunity against the antigen PyMSP1 19 with increased production of antibodies by an external magnetic stimulus. The immunization with the complexes induced dendritic cell maturation through the upregulation of CD86 expression in the spleen. The presence of secondary effector T cell-mediated immune responses was noted as CD4 helper T cells (Th). The complexes induced antigen-specific Th1 and Th17 cells (Al-Deen et al., 2017). In addition, a non-inflammatory delivery system based on polystyrene nanoparticles (PSNPs) complexed with antigen MSP4/5 (surface proteins of *P. falciparum/P. vivax* merozoites) and Freund’s adjuvants and alum was tested in a mice model. The non-inflammatory adjuvants associated with PSNPs induced a Th1 immune response acting similarly to a vaccine. The PSNPs-MSP4/5 conjugates induced immune responses by Th1 and Th2, and antibody subclasses IgG1, IgG2a, and IgG2b. The response using adjuvants was even higher. IL-4-associated with Th2 responses induced IgG1 antibodies, and IgG2 antibodies were related to Th1 responses and dependent on IFN-γ. Immunization protection against malaria blood-stage infection may depend on IFN-γ production (Wilson et al., 2019).

Herein, we aimed to discuss the potential application of nanotechnology in developing new antimalarial drugs and the gap between preclinical and clinical studies based on nanotechnology. Table 3 presents the potential and limitations of nanotechnology.

**TABLE 3:** Main polymeric drug nano vehicles.

Nanosystems	Application potential	Application limitation	References
Dendrimers	Presence of internal spaces to encapsulate drugs; external part with functional groups for conjugation and targeting of therapeutic agents; low polydispersity index; ideal for malaria treatment	High cost; possibility of loss of encapsulated substance	Dias et al. (2020), Hu et al. (2020), Nosrati et al. (2018), Sahoo et al. (2020), Zhang et al. (2020)
Nanogels	Encapsulate therapeutic agents in the gel matrix by diffusion; transport and encapsulate hydrophilic and hydrophobic drugs; sustained drug release	Rapid release of therapeutic agents; therapeutic agent leakage	Dawre et al. (2018), Owonubi et al. (2018), Rashidzadeh et al. (2021)
Micelles	Internalization of hydrophobic drugs with protection from degradation; control of drug release rate; decreased side effects and cytotoxicity	Micelle stability *In vivo* during blood circulation due to dilution; decreased o tempo de half-life; therapeutic agent leakage	Ramazani et al. (2018), Ismail et al. (2019), Rashidzadeh et al. (2021)
Drug polymer conjugate (PDC)	Direct drug delivery to the target site; sensitive to the pH of the exposed environment for the release of therapeutic agent	May cause hemolysis	Nosrati et al. (2018), Rashidzadeh et al. (2021)
Liposomes	Ability to encapsulate hydrophobic and hydrophilic drugs; prevents degradation and promotes the delivery of a therapeutic agent to a specific site	Structural instability; drug leakage; opsonization	Fotoran et al. (2019), Apolinário et al. (2020), Rashidzadeh et al. (2021), Huang (2020)
Nanoparticles	Drug targeting to take the drug to the exact site of action, increasing bioavailability, concentration, and elimination of drugs	High cost	Kannan et al. (2019), Martí Coma-Cros et al. (2019), Panzarini et al. (2018), Pestehchian et al. (2020), Wang et al. (2021), Zhang et al. (2020), Wilson et al. (2019)
Metallic and magnetic nanoparticles	Metallic and magnetic nanoparticles containing antigens of the sexual state and/or drug delivey and induction of malaria transmission-blocking immunity	Few studies on how size, shape, and surface charge affect the efficiency of immunogenicity	Kumar et al. (2015), Al-Deen et al. (2017), Powles et al. (2020)

Source: Recent advances in targeting malaria with nanotechnology-based drug carriers (Rashidzadeh et al., 2021).

### Recent studies on nanotechnology in malaria treatment

**FIGURE 5:**
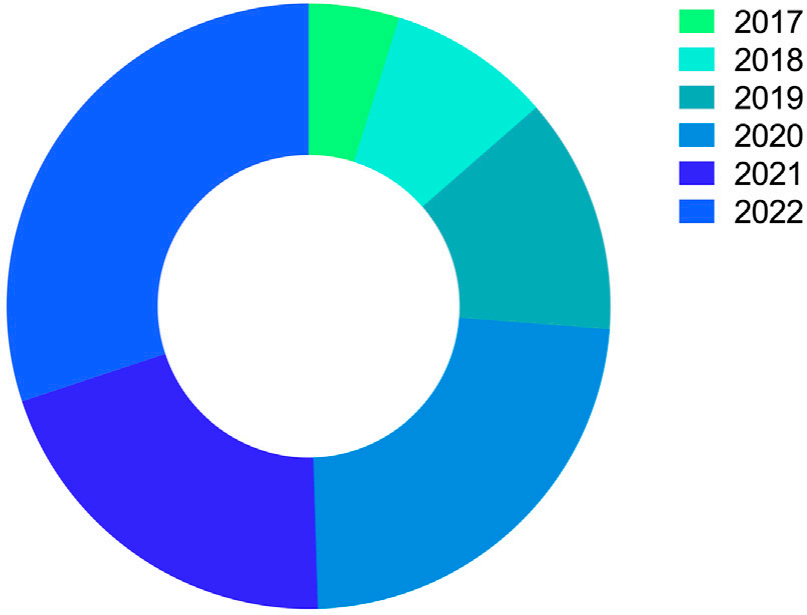
Currently published articles on nano-formulated therapies against malaria. The illustration shows that data recorded from 2017 to 2022 were evaluated and retrieved from the National Library of Medicine USA databases (MEDLINE∕PUBMED—NLM), Scientific Electronic Library Online (SciELO), and Google Scholar (Google Scholar).

The number of publications on nanoparticles in treating malaria over time is increasing. For instance, a search on the National Library of Medicine USA databases found only one publication from 1990 to 2000 and 8 from 2001 to 2010. Herein, a total of 103 publications that address the study of nanoparticles in malaria treatment were analyzed. They are divided yearly into the following numbers: 2017-5 articles, 2018-9 articles, 2019-13 articles, 2020-24 articles, and 2021-21 articles (Figure 5). In 2022, a growing number of publications that address studies related to nanoparticles in treating malaria will be noticeable. In 2022, 31 publications related to this field are available for consultation.

From 2019 to 2022, multiple studies based on nanotechnology application in malaria treatment reaffirmed positive conclusions, suggesting the potential of nanosystems. Conversely, only a few ongoing clinical trials based on drug delivery for malaria treatment have been reported. In the last decade, dendrimers have caught interest for several biological applications, such as drug and gene delivery and diagnostic[S1] imaging agents (Araujo et al., 2018; Dias et al., 2020). Dendrimers are quasi-spherical, nanometer-sized, tree-like polymeric macromolecules containing many reactive functional subgroups, branches, and protected interior spaces (Chauhan, 2018; Sherje et al., 2018). The ramifications of the nanostructure generate layers, or “generations”, which characterize the size of the cavity, being the most reported in the first, second, or third generations (one, two, or three layers). These nanosystems can carry hydrophilic and hydrophobic drugs, as they have many functional groups in the periphery (Chis et al., 2020). Furthermore, they target drugs to specific sites and favor cellular uptake, mainly by endocytosis and passive diffusion (Russier et al., 2015; da Silva Santos et al., 2016; Akbarzadeh et al., 2018).

Preclinical studies using nanotechnology to treat malaria are summarized in Table 4. For instance, ultrasmall gold nanoparticles based on glucose or nanogold clusters (Glc-NCs) were developed for use in the intraerythrocytic stage of *P. falciparum* (*in vitro*) in both phases of parasitic development (asexual and sexual) without nonspecific connections or red blood cell lysis. Glc-NCs loaded with ciprofloxacin showed 50% higher antibiotic effect than that of free drug, demonstrating its therapeutic potential (Varela-Aramburu et al., 2020). Silver nanoparticles (AgNPs) synthesized using *Artemisia* sp leaf extract showed high antimalarial activity in *P. falciparum* cultures (Avitabile et al., 2020). AgNPs from *Salvia officinalis* leaf extract showed hepatoprotective and antiplasma effects, reducing parasitemia and hepatic oxidative stress markers in an experimental malaria model (Metwally et al., 2021). Moreover, AgNPs from *Indigofera oblongifolia* leaf extract decreased parasitemia and showed anti-inflammatory, antioxidant, and anti-apoptotic effects in mice infected by *Plasmodium chabaudi* (Dkhil et al., 2019).

**TABLE 4:** Summary of different drug-loaded micro- and nanoparticles reported from 2019 to 2021.

Nanoparticles Microparticles	Drug used	Types of formulation	Kind of study	References
Polymeric microparticles	Primaquine	Intravenous solution	Preclinical	Da Silva De Barros et al. (2021)
Nanoemulsions	Primaquine; dihydroartemisinin; thiazoline; azacarbazole^®^	Intravenous solution; intragastric gavage	Preclinical *In vitro^®^*	Umeyor et al. 2019, Silva and Cardoso (2020), Jaromin et al. 2021, Wu et al. 2021
Lipid nanoparticles/lipid carriers	Primaquine (*in vitro/ex vivo*) dihydroartemisinin; artesunate; artemether; lumefantrine; artefenomel (OZ439) (*in vitro*); curcumin	Intranasal administration; transdermal nanogel	Preclinical	Salim et al. (2019), Umeyor et al. (2019), Ghosh and Banerjee (2020), Agbo et al. (2021), Guo et al. (2021), Nnamani et al. (2021), Wu et al. (2021)
Unilamerlar vesicles	Primaquine	Intravenous injection	Preclinical	Al Fayes et al. (2021)
Liposomes/liposomes; peguilated	Primaquine; chloroquine; decoquinate ( *in vitro*)	Intravenous solution	Preclinical	Duan et al. (2020), Marwah et al. (2020), Miatmoko et al. (2021)
Multilamellar nanoliposome	Chloroquine	Intraperitoneal injection	Preclinical	Fotoran et al. (2019)
Nanodendrimer/globular nanodendrimer	Chloroquine; curcumin	1HNMR spectroscopy (*in vitro*)	Preclinical	Elmi et al. (2021)
Dextran nanoparticle	Chloroquine diphosphate; chloroquine	Particle nanosuspension dialysis (*in vitro*)	Preclinical	Kashyap et al. (2018)
Metal nanoparticles (gold, silver, ferrite, iron oxide)	Ciprofloxacin (*in vitro*); artemisinin; *Salvia officinalis*; *Indigofera oblongifolia*; artesunate	Orally inoculated; intraperitoneal injection	Preclinical	Avitabile et al. (2020), Dkhil et al. (2019), Kannan et al. (2019), Varela-Aramburu et al. (2020), Zhang et al. (2020)
Decorated nanoparticle/nanosphere	Artemisinin	Intravenously administered	Preclinical	Gérard Yaméogo et al. (2020)
Conjugated micellar nanocomplex/nanomicelle	Artemisinin; artesunate; pyrimethamine; pluronic^®^F127	Intravenously administered; oral solution; intragastric gavage	Preclinical	He et al. (2021), Ismail et al. (2019), Manconi et al. (2019), Martí Coma-Cros et al. (2019), Pestehchian et al. (2020)
Conjugated nanocapsules/polysorbate	Artesunate; quinine	Intraperitoneal injection; oral solution	Preclinical	Ismail et al. (2019), Michels et al. (2019), Moreira Souza et al. (2020)
Lyophilized nano suspension	Atovaquone; artesunate; artemether	Oral gavage; intradermal route; oral solution	Preclinical	Kathpalia et al. (2020), Volpe-Zanutto et al. (2021)
Pasty polymer	Artemisone	Subcutaneously injected	Preclinical	Golenser et al. (2020)
Immunoliposomal nanovector	Atovaquone; pyronaridine	Oral delivery	Preclinical	Biosca et al. (2019)
Phospholipid complex	Lumefantrine	Subcutaneous or intramuscular injection	Preclinical	Kaur et al. (2021)
Nanogel	Artemether	Nanogéis ART transdérmicos	Preclinical	Nnamani et al. (2021)
Zein nanoparticles	Artemether	Intravenously administered	Preclinical	Boateng-Marfo et al. (2021)
Flash nanoprecipitation	Lumefantrine; artefenomel (OZ439)	Oral delivery	Preclinical	Ristroph et al. (2019), Wang et al. (2021)
Neutral nanoparticle	Curcumin	Oral delivery	Preclinical	Biosca et al. (2021)
Nutrissomes	Curcumin	Oral solution	Preclinical	Manconi et al. (2019)
Gold nanoparticle	Antigens delivery	Subcutaneous or intramuscular injection	Preclinical	Kumar et al. 2015
Superparamagnetic nanoparticles	Magnetic nanovector	Subcutaneous or intramuscular injection	Preclinical	Nawwab al-deen et al. (2017)
Polystyrene nanoparticles	Non-inflammation-inducing polystyrene nanoparticle delivery system	Subcutaneous or intramuscular injection	Preclinical	Wilson et al. (2019)

Source: https://pubmed.ncbi.nlm.nih.gov/.

Hollow nanoparticles of mesoporous ferrite (HMFNs) with ferromagnetic properties were prepared using artemisinin and targeted to hemozoin produced by *P. falciparum* in infected erythrocytes. It acted as a targeted delivery system, increasing the local concentration of ART through its association with heparin, suggesting its potential in antimalarial therapy (Wang et al., 2021). Surface-loaded nanoparticles containing ART in two types of formulations (nanospheres and nanocapsules) had longer elimination half-life than that of an ART solution in ethanol, suggesting their potential as intravenous antimalarial agents (Gérard Yaméogo et al., 2020). Furthermore, a stable ART-based injectable nanocomplex, composed of dimorphic artesunate-choline (dACC) micelles coated with hyaluronic acid (HA), demonstrated safety and antimalarial activity in *in vitro* and *in vivo* experiments compared to those of free artemisinin/artesunate, suggesting it as a promising injectable alternative to the traditional artesunate (ATS) used in malaria treatment (He et al., 2021).

*In vitro* and *in vivo* studies have shown that lipid nanoemulsions with modified surface are ideal carriers of dihydroartemisinin (Umeyor et al., 2019). Ester-linked dihydroartemisinin trimer (DHA 3) prepared as self-assembled nanoparticles (DHA 3 NPs) demonstrated superior antimalarial effects compared to that of control in a murine experimental model, with improved cure rate and survival time and reduced recurrence rate in experimental animals (Guo et al., 2021). ATS fortified with iron oxide-coated nanoparticles showed increased cytotoxicity and selective damage to *P. falciparum* in a murine experimental model, suggesting a potent antimalarial agent against artemisinin-resistant malaria (Kannan et al., 2019). Nanostructured lipid carriers (NLCs) loaded with ATS for intranasal administration (ATS-NLCs) showed great potential as an alternative to parenteral administration in hard-to-reach regions, simplifying the treatment of severe and cerebral malaria in adults and children (Agbo et al., 2021). The nanocapsules based on artesunate-heparin conjugate (ATS-HEP-NCPs) were used as drug delivery vehicle for intracellular release; *in vitro* and *in vivo* experiments showed increased stability and improved pharmacokinetic properties (Ismail et al., 2019). In a murine experimental model, the triple combination containing atovaquone in a lyophilized suspension of proguanil-artesunate demonstrated prophylactic efficacy.

In addition, the use of double (atovaquone/proguanil) and triple (atovaquone/proguanil/artesunate) combination formulations resulted in a complete cure (Kathpalia et al., 2020). The injectable pasty polymer, formulated for controlled release of artemisinin, reduced parasitemia and severe symptoms in cerebral malaria and increased survival of animals, with an increase in the half-life of the drug compared to that of free drug in an experimental murine model (Golenser et al., 2020). The nanovector immunoliposomal encapsulating the antimalarials pyronaridine and atovaquone, which block the development of gametocytes through targeting glycophorin *in vitro*, presented significantly higher efficacy than that of the free forms (Biosca et al., 2019).

The improved solubility of lumefantrine (LUM) in an aqueous medium by using lumefantrine phospholipid complex (LMF-PC) enhanced antimalarial activity in a murine model (Kaur et al., 2021). The matrices of microneedle (MN) containing ART (MN-ART) and LUM (MN-LUM) in nanosuspensions, applied as intradermal devices, showed increased solubility of drugs and potential as an alternative treatment for malaria in endemic regions with scarce resources (Volpe-Zanutto et al., 2021). Transdermal artemether nanogel (TMT) as NLCs have demonstrated good antimalarial properties in *in vitro* and *ex vivo* skin permeation experiments. They offered 100% cure and negligible side effects when two adhesives were applied per week at a lower dose than that of free TMT (Nnamani et al., 2021). Zein nanoparticles loaded with TMT showed good encapsulation efficiency, reduced hemolysis, and prolonged therapeutic effect compared to that of free TMT (Boateng-Marfo et al., 2021). Caprol-based NLCs were prepared to improve the bioavailability of artemether/lumefantrine antimalarials; they showed improved oral bioavailability, antimalarial activity, and potential compared to those of free antimalarials (Akpa et al., 2020). In addition, nanocapsules (NC-ATM) showed decreased neurotoxic and cardiotoxic effects in mice infected with *Plasmodium berghei*, a safe alternative to TMT for the treatment of malaria (Moreira Souza et al., 2020). Flash nanoprecipitation (FNP) is a technique that enables the production of nanoparticles from laboratory-scale to industrial pilot-scale. Lumefantrine was processed by FNP to obtain 200 nm nanoparticles with increased bioavailability and dissolution kinetics suitable for industrial-scale production (Ristroph et al., 2019). The technique (FNP) was employed for formulation of the substance OZ439 (artefenomel), which is active against drug-resistant malaria, and the formulation showed potential as a single-dose cure. Powder formulations using spray dryer is appropriate for industrial-scale production. *In vitro* tests have shown that the formulation of the antimalarial OZ439 in a single-dose oral form had good stability against humidity and temperature (Ristroph et al., 2019). The oral bioavailability of OZ439 and the adjustment of interactions by selecting alternative systems such as milk-like lipid nanoparticles or powder systems can provide different possibilities for enhanced solubilization and absorption for this drug (Salim et al., 2019).

Neutral zwitterionic amphiphiles forming nanoparticles (PBMA-MESBMA) using curcumin and targeted to infected erythrocytes showed effective and faster antimalarial activity than that of free curcumin in an animal model (Biosca et al., 2021). Nutrisomes (phospholipid vesicles + Nutriose^®^ FM06) were modified to obtain new systems with increased efficacy of curcumin as an antimalarial agent after oral administration. Eudragit^®^ L100 (EUD) was added to these vesicles to improve their *in vitro* and *in vivo* performance, which showed an increased ability to neutralize oxidative stress in intestinal cells and increased survival of infected mice compared to controls treated with free curcumin (Manconi et al., 2019). The nanocapsules (NCP80) and nanospheres (NSP80) of polysorbate 80 and nanocapsules (NCEUD) and nanospheres (NSEUD) of Eudragit RS 100 containing quinine were evaluated for their effects on the surface characteristics and antimalarial efficacy *in vitro* and *in vivo*. Cationic and anionic nanocapsules have been developed to deliver quinine to erythrocytes using Eudragit RS 100. An improvement in antiplasmodial efficacy was observed along with altered characteristics of the cationic nanocapsules with quinine such as protection against light and improvement of quinine partition coefficient *in vitro*. Improvement in biodistribution of quinine by NCEUD and increase in the half-life of elimination of quinine *in vivo* suggest it as a potential alternative for the treatment of malaria (Michels et al., 2019). The nanoformulation of curcumin in combination with the compound benzothiophene 6 (3-bromine-N-(4-fluorobenzyl)-benzo[b]thiophene-2-carboxamide) showed sustained release of curcumin, increased stability and solubility in aqueous medium, and antimalarial activity in in *vivo* and *in vitro* experiments (Ghosh and Banerjee, 2020).

The nanoemulsion delivery system of azacarbazoles, derived from carbazole (9 H-carbazole) with a concentrated form of ethyl esters of polyunsaturated fatty acids n-3 and n-6, provided evidence of increased antiplasmodial activities *in vitro,* without cytotoxic effects against mammalian cells, showing rapid absorption after intragastric administration (Jaromin et al., 2021). In addition, thiazoline nanoemulsion (10-(4,5-dihydrothiazol-2-yl)thio)decan-1-ol), a synthetic compound similar to 3-alkylidene marine alkaloid being reported as an antimalarial substance, reduced *in vivo* parasitemia and increased antimalarial activity *in vitro* (Silva and cordoso., 2020). The 407 poloxamer nanomicelles loaded with pyrimethamine showed potent antimalarial activity and lower liver damage in a murine experiment than that of the free compound, indicating potential for adaptation as an antimalarial formulation (Pestehchian et al., 2020). *In vitro* and *in vivo* experiments have shown that hybrid dendritic-linear-dendritic block copolymer mycelial transporters based on Pluronic^®^ F127 (HDLDBC-bGMPA) are promising for the development of future antimalarial drugs aimed at penetrating erythrocytes (<30 nm) infected by *Plasmodium* sp (Martí Coma-Cros et al., 2019). *In vitro* and *in vivo* experiments of artelinic acid (AD) derivatives formulated as liposomes (ADLs) showed superior antimalarial efficacy compared with that of the control groups. Pharmacokinetic results of ADLs demonstrated the slowest elimination and highest total concentration in plasma, showing potential for the treatment of malaria (Duan et al., 2020).

Furthermore, primaquine polymeric microparticles (PPM) in *in vivo* experiments using a murine model showed partial efficacy and protection against parasite development compared with that of free primaquine, suggesting the potential of this drug delivery system for the treatment of malaria (da Silva de Barros et al., 2021). Three drug carriers based on lipid formulations loaded with primaquine, solid lipid nanoparticles, nanoemulsions and nanostructured lipid carriers were developed and evaluated; all lipid formulations could successfully protect erythrocytes from cell lysis caused by free primaquine (Wu et al., 2021). Phospholipid-free unilamellar vesicles (PFSUVs) composed of Tween 80 and cholesterol were evaluated in experimental models *in vitro* and *in vivo*. They effectively delivered primaquine to the liver, selectively targeted hepatocytes, and reduced erythrocyte uptake compared to that of free primaquine, leading to reduced erythrocyte toxicity (Al Fayes et al., 2021). *In vivo* experiments with PEGylated liposomes containing primaquine and chloroquine for the treatment of malaria in the hepatic stage demonstrated that liposomal membrane fluidity was greatly affected by the double burden of primaquine and chloroquine drugs, and additional studies related to stabilization of these liposomes are needed (Miatmoko et al., 2021). Decoquinate (DQN) is a molecule that can potentially function as a substitute for active primaquine against malaria in the hepatic stage was directed to hepatocytes infected by the parasite using a liposomal transporter system *in vitro* and *in vivo*. The study concluded that glycyrrhizic acid receptors participated in the targeted delivery of DQN liposomes to the hepatocytes (Marwah et al., 2020). Multilamellar nanoliposomes stabilized by hydrogen bonds containing chloroquine targeted to infected erythrocytes showed antiparasitic effect superior to that of free chloroquine in *in vitro* and *in vivo* studies, being permissive for smaller and larger molecules (Fotoran et al., 2019). The loading of chloroquine in G2 nanodendrimers showed antiplasmodial activity and decreased toxicity of structured nano chloroquine compared to that of free chloroquine, indicating that this compound is an effective antiplasmodic agent *in vivo* in a murine model (Elmi et al., 2021). 1H NMR spectroscopy was used to study the effect of anionic linear globular dimerized nanocomplexes based on curcumin loaded with chloroquine G2 and antiplasmodial effect against *P. falciparum in vitro* (Elmi et al., 2021). In addition, dextran NPs loaded with chloroquine diphosphate (CHQ-DEX-NPs) were developed to overcome resistance of *P. falciparum* to chloroquine *in vitro*. CHQ-DEX-NPs could trigger the parasite’s apoptotic pathway by accumulating in food vacuoles and were found safe for parenteral administration (Kashyap et al., 2018). The biocompatible and biodegradable nanoparticles of chitosan-tripolyphosphate-chloroquine triggered elimination of multidrug-resistant parasites through redox action, modulating pro- and anti-inflammatory responses, suggesting a new approach to treat multidrug-resistant malaria. The CS-TPP CQ nanoparticles killed the parasite and diminished the production of the pro-inflammatory cytokines TNF-α and IFN-γ, and increased the anti-inflammatory cytokines IL-10 and TGF-β (Das et al., 2021). The incorporation of proteins or peptides of interest can occur both during the preparation of the NP (e.g., antigens), and through their complexation/conjugation on the nanocarrier surface (e.g., plasmids or antibodies). For example, Cherif et al. (2011) developed polymeric NPs formed by pDNA complexed with PEI (cationic polymer) and PLA (biocompatible polymer), which were tested *in vivo* against *P. yoelii* in different administration routes (IV, SC, and IP) in mice. They observed an increase in the immune response regarding the levels of IgG, T cells (CD4^+^ and CD8^+^), IFN-γ and IL-12. Another study carried out by Collins et al. (2017) developed NPs conjugated to the surface antigen of the circumsporozoite protein CSP-hepatitis B, acting in the pre-erythrocyte stage of infection by *P. falciparum*. Moderate levels of protection were induced through the immune response mediated by CSP-specific antibodies. In addition, this vaccine was immunogenic (cellular and humoral immune response) at low doses. Also, when administered with Abisco-100 and Matrix-M adjuvants, it induced protection against transgenic sporozoites (Collins, 2017).

Articles have discussed the multi-component vaccination to challenge both humoral and cellular immunity and adjuvants as the best strategy to reach all stages of malaria.

The association of polymeric and lipid NPs is also described, as reported by Kumar et al. (2015). Nanoemulsion containing squalene, chitosan/PLGA NPs and CHrPfs25 were developed and evaluated against *P. falciparum.* Results regarding the functional immunogenicity generated by CHrPfs25 are promising. Authors found IgG from mice immunized with different GN-particles with highly potent blocking activity regardless of IgG isotype differences. Most studies in this article suggest nanoformulations as drug delivery systems, indicating their potential for the treatment of malaria (Table 4).

### Limitations of nanotechnology in malaria treatment

Nanotechnology has enormous potential for the development of new drugs against malaria, although there are limitations. Despite significant advantages of nano-pharmaceutical release systems, factors such as high preparation cost, interaction with biological components, difficulty in production scale up depending on the development method, failure to define the appropriate route of administration, and failure in therapeutic reproducibility demotivated researchers to lead preclinical research for clinical application (Neves Borgheti-Cardoso et al., 2020; Rashidzadeh et al., 2021).

The cost of producing nanosystems is a primary concern for their use in treating diseases such as malaria. The large-scale production of nano drugs to provide access in malaria predominant regions is difficult due to financial, logistical, and political issues, among other limiting factors (Feng et al., 2019; Ristroph et al., 2019). In addition, majority of malaria-affected population is concentrated in developing countries, which have low resources invested in health and medical care. This panorama of resource scarcity failed to foster industry interest in the development of new antimalarial medicines on a large scale because of low financial returns (Ristroph et al., 2019; Volpe-Zanutto et al., 2021).

Despite considerable volume of studies based on nanotechnology application for the treatment of malaria, it is possible to perceive a difference between the number of preclinical and clinical studies, which are lower. Preclinical studies that employ nanoparticles in other diseases, such as cancer, tumors, and Alzheimer’s disease, present more signifying results and often involve theranostic nanomedicine (Gandhi et al., 2020; Abdolmaleki et al., 2021). High cost of preparation, drug administration routes, bioaccumulation, toxicity, and interaction with biological components are some critical issues to be resolved to contemplate the success of new nanotechnological strategies for the treatment of malaria (Apolinário et al., 2020; Rashidzadeh et al., 2021).

To overcome the clinical failures of antimalarial therapy, developing new medicines is urgent and necessary. However, developing a new drug is expensive, complex, and time-consuming. In this case, strategies that increase the therapeutic efficacy of current conventional antimalarial drugs and reduce their toxicity are promising alternatives. For that, nanotechnology can be considered an approach to solving these inherent limitations, such as the low water solubility, biodegradability, bioavailability, and parasite resistance (Jawahar et al., 2019; Puttappa et al., 2019; Rashidzadeh et al., 2019; Abazari et al., 2020; Bagheri et al., 2020). Several organic, inorganic, and hybrid nanometric systems were discussed in this review and offer many alternatives as drug delivery systems for antimalarial drugs (Table 3). Therefore, these efforts must be continued to accelerate the clinical application of these systems to treat malaria.

Some companies have explored the potential of dendrimers. For instance, Starpharma developed the DEP^®^ platform for drug delivery applied to antitumor drugs. Or diagnostic kits component as 3DNA^®^ from Gemisphere, or transfection agents such as Polyfect^®^ and Superfect^®^ from Qiagen (Dias et al., 2020). In addition, according to information from the American repository ClinicalTrial.gov, some clinical trials evaluate drugs associated with dendrimers for treating cancer, bacterial infection, or COVID-19 (Caminade, 2022). However, up to date, there are no dendrimer-based medicines to treat malaria, either under clinical evaluation or commercialization.

## Conclusion

Malaria is a disease affecting millions of people worldwide. Despite the current treatments, resistance to antimalarial drugs has increased. Advances in nanotechnology for the development of new drug delivery systems are promising and being increasingly studied in preclinical tests, with significant and instigating results. However, no nano-formulated antimalarial drug is currently available for clinical use.

New approaches to malaria treatment are urgently needed owing to the complexity of the biological cycle of the parasite, spread of the disease in conditions where sanitary and social conditions are precarious, and reports of resistance of the parasite to conventional therapies. The interaction between academic research, pharmaceutical industry, and political leaders is the most promising path for the development of new drugs and nanostructured drug delivery systems.
